# Effect of linear alkylbenzene sulfonate on the uptake of microcystins by *Brassica oleracea* and *Solanum tuberosum*

**DOI:** 10.12688/f1000research.125540.2

**Published:** 2024-01-26

**Authors:** Glynn Pindihama, Mugera Gitari, Ntakadzeni Madala

**Affiliations:** 1 Department of Geography & Environmental Sciences, University of Venda, Thohoyandou, Limpopo Province, 0950, South Africa; 2 Department of Chemical Sciences and Technology, Technical University of Kenya., Nairobi, Kenya, 00200, Kenya; 3 Department of Biochemistry and Microbiology, University of Venda, Thohoyandou, Limpopo Province, 0950, South Africa

**Keywords:** Linear alkylbenzene sulfonate, Microcystins, Brassica oleracea, Solanum tuberosum, combined effects

## Abstract

**Background:**

Globally, hypereutrophic conditions in major water reservoirs used for irrigation purposes, promote the co-existence of cyanotoxins and other pollutants such as linear alkylbenzene sulfonate (LAS). LAS is known to alter the permeability of membranes and promote the uptake of other pollutants by plants. In light of the potential human health risks and prevailing hypereutrophic conditions in some catchments in South Africa, we investigated the combined effects of LAS and microcystins (MCs) on food plants when cyanobacteria infested water is used to irrigate terrestrial crops.

**Methods:**

To understand the potential risks, pot-culture experiments were conducted to assess the effect of LAS on the accumulation of MCs in *Brassica oleracea* (cabbage) and *Solanum tuberosum* (potato) plants. The plants were watered with dam water containing 3.48 mg L ^-1^ of the LAS (sodium dodecyl sulfate) and MCs (MC-LR: 10.47 ± 3.879; 6.158 ± 4.127 for MC-RR and 8.160 ± 2.544 for MC-YR μg L ^-1^) for 20 days.

**Results:**

The presence of LAS, at environmentally relevant concentrations in the irrigation water, did not enhance the uptake of MCs in the two plants, as demonstrated by statistically insignificant differences in the means of the treatments (with and without LAS). In addition, the presence of LAS, high pH, electrical conductivity (EC), and cyanotoxins in the water did not affect the total chlorophyll or the well-being of the plants. However, in some cases the levels of MCs bioaccumulated by the two plants exceeded the WHO recommended tolerable daily intake (TDI).

**Conclusions:**

These findings imply that the tested levels of LAS and MCs did not have any synergic effects on the two plant species, but irrigating food crops with such water still poses a human health risk.

## Introduction

Globally, the incidents of harmful algal blooms (HABs) have been on the rise in the last few decades. Such increases have been linked to growing urbanization and the resultant increase in nutrients loads into the aquatic environment, higher atmospheric temperatures and salinity, all of which are worsened by human-induced climate change ( Zhao *et al*., 2013; Howard *et al*., 2017; Díez-Quijada *et al*., 2018). South Africa is a semi-arid country with limited water resources and thus relies on surface waters for irrigation, domestic and industrial uses. The country mainly relies on man-made reservoirs for potable and irrigation water supplies, unfortunately, such surface water sources are increasingly being contaminated by cyanobacteria and cyanotoxins which can bioaccumulate in plant tissue when such water is used for irrigation. This makes the consumption of crops and vegetables irrigated with the contaminated water a potentially dangerous route for human exposure to different cyanotoxins.

There are numerous types of cyanotoxins that have been reported and documented, but the liver toxins, microcystins (MCs), are the most commonly occurring toxins in freshwater, hence the most widely studied. Contaminated drinking and recreational water are known to pose immediate human health threats, but recent studies have demonstrated food consumption as another exposure route, since cyanotoxins can accumulate in animal and plant tissues ( Manubolu *et al*., 2018). Thus, drinking water and the consumption of contaminated fish, crops and other food supplements are now recognized as routes for human oral exposure ( Díez-Quijada *et al*., 2018).

Due to the recognition of the potential health threats that could be posed by cyanotoxins, the World Health Organization (WHO) has set thresholds for MCs in both drinking water and food ( Howard *et al*., 2017). The WHO has set a provisional tolerable daily intake (TDI) of 0.04 μg kg ^-1^ of body mass (b.m.) for MCs and 1 μg L ^-1^ as the upper limit for MC-LR in drinking water ( Díez-Quijada *et al*., 2018; Yao *et al*., 2019). In plants, MCs are also known to have numerous damaging effects such as reducing photosynthesis, causing necrosis of tissues, inducing oxidative stress, reducing productivity of crops, and causing economic losses ( Campos *et al*., 2021).

Linear alkylbenzene sulfonates (LAS) on the other hand, belong to a group of anionic surfactants commonly used in domestic and industrial processes ( Wang *et al*., 2015). Anionic surfactants are a common ingredient in detergents due to their simple synthesis and low cost ( Pierattini *et al*., 2018). LAS is known to alter membrane permeability and in turn affect the toxicity and accumulation of other toxins such as cyanotoxins in organisms ( Wang *et al*., 2012). LAS find their way into the aquatic environment through the discharge of untreated and treated wastewater. LAS elimination in the aquatic environment occurs via adsorption onto particles of sediments ( Thomas *et al.*, 2017) and biodegradation ( Wang *et al*., 2012). The biodegradation however, occurs very slowly in anaerobic and anoxic environments and this leads to their accumulation under such conditions in water ( Wang *et al*., 2012).

This makes hypereutrophic lakes and reservoirs ideal environments for the co-existence of toxic cyanobacteria and LAS since the excessive growth of cyanobacteria in eutrophic lakes consumes oxygen and their eventual death and degradation makes water bodies anoxic and anaerobic ( Wang *et al*., 2012). Previous studies reported enhanced MCs uptake by plants in the presence of LAS ( Wang *et al*., 2012). Furthermore, Wang *et al*. (2015) also found increased production of MCs by *Microcystis aeruginosa* in the presence of LAS.

In South Africa, the Crocodile (West) Marico Water Management Area (WMA) which covers parts of Gauteng and Northwest Provinces, houses dams such as the Hartbeespoort, Rietvlei, Roodeplaat, and Bospoort Dams. For many decades these dams have been classified as hypertrophic ( van Ginkel, 2004) and the co-existence of pollutants such as LAS, cyanotoxins, and other pollutants is thus likely in these reservoirs. This raises the questions whether the co-existence of these pollutants increases human health risks to consumers of the irrigated crops, particularly with the water derived from these hypertrophic dams being used for irrigation purposes.

The aim of this study was to assess the effect of anionic surfactants (LAS) on the accumulation of three MC congeners (MC-LR, MC-RR and MC-YR) in *Brassica oleracea* (cabbage) and *Solanum tuberosum* (potato) plants using environmentally realistic concentrations of the pollutants. Water used in the study was collected from reservoirs in Crocodile (West) and Marico catchment, considering the prevailing hypertrophic conditions in the catchment. 

## Methods

### Materials and reagents

A field survey was conducted from the 23 ^rd^ to the 25 ^th^ of June 2019 and again from the 14 ^th^ to the 16 ^th^ of September 2019 to collect field water to be used for the experiments. The water was collected from canals and farm dams from the Roodeplaat and Hartbeespoort dam sites. Total dissolved solids (TDS), EC, pH, and turbidity of the water were monitored *in-situ* and anionic surfactants, chlorophyll- *a*, microcystins (MCs), and cations were measured *ex-situ.* The water was kept frozen at −20 ^o^C until required. The LAS used in this study was sodium dodecylbenzene sulfonate (SDS) (CAS No. 25155-30-0; molecular mass 348.48 g mol ^-1^; and chemical formula C _18_H _29_NaO _3_S acquired from BYMAZ Pty Ltd, Johannesburg, South Africa).

### Pot-culture experiment design

The *B. oleracea* seeds were purchased from NTK Agricultural Products & Services (S.A) and the *S. tuberosum* seeds were purchased from Livingseeds Heirloom Seeds (Pty) Ltd Midvaal, Gauteng (S.A). The *S. tuberosum* seeds were first washed with distilled water before being planted in 18 L (350 mm) plastic pots filled with non-contaminated soil. The *B. oleracea* seedlings were produced and pre-grown in plastic trays with non-contaminated soil. The soil used in this study was collected from the agricultural farm at the University of Venda. The farm lies in the Lowveld climate and has well-drained deep red soils mostly dominated by clay and falls in the Hutton classification, which is the same as the Rhodic Ferralsol ( Mabasa, 2019). Regarding the main nutrients, phosphorus (P), potassium (K), total nitrogen (N) and organic matter, the soil contained 25.86 (mg kg ^-1^); 184 (mg kg ^-1^); 0.079% and 2.07%, respectively, all of which indicated healthy soils for plant growth. The soil was collected from a depth of 0-50 cm, and approximately 15 kg of the soil was placed into 18 L (350 mm) plastic pots for the experiments and treated with 0.375 g (per kg of soil) of Protek General Fertilizer with N:P:K (%) 2:3:2 (14) before introducing the plants.

To investigate the effect of LAS on MCs uptake and accumulation in *B. oleracea* and *S. tuberosum*, the plants were watered daily with water collected from the Roodeplaat Dam. The Roodeplaat Dam is renowned for its heavy presence of the cyanotoxin, microcystin and seasonal blooms of cyanobacteria, with the *Microcystis* spp. reported as the most dominant species ( Modley *et al.*, 2019). The water had a mean concentration ± standard deviation of MC-LR: 10.47 ± 3.879; 6.158 ± 4.127 for MC-RR, and 8.160 ± 2.544 for MC-YR.

In order to maintain approximately constant concentrations of the LAS (3.4 mg L ^-1^), fresh media were prepared daily just before watering the plants. After 5 days and 20 days of exposure, the accumulations of MCs in edible parts of *B. oleracea* (leaves) were measured ( Table 1). For *S. tuberosum,* the accumulated MCs in the tubers were assessed only after 20 days of exposure, since the seeds take longer to sprout. Leaves were also harvested from both plant species on the 20 ^th^ day of exposure for total chlorophyll determination.

**Table 1.** Design of the experiment and sampling intervals.

Treatments	Parameters monitored
**Treatment 1 (T1)**: Control: Milli-Q water with no contaminants	After 5 days of exposure: MCs in *B. oleracea* After 20 days of exposure: MCs and total chlorophyll in *B. oleracea* & *S. tuberosum*
**Treatment 2 (T2):** Raw dam water containing MCs (MC-LR: 10.47 ± 3.879 μg L ^-1^; MC-RR: 6.158 ± 4.127 μg L ^-1^; MC-YR: 8.160 ± 2.544 μg L ^-1^)	After 5 days of exposure MCs in *B. oleracea* After 20 days of exposure: MCs and total chlorophyll in *B. oleracea* & *S. tuberosum*
**Treatment 3 (T3):** Milli-Q water with 3.4 mg L ^-1^ LAS	After 5 days of exposure: MCs in *B. oleracea* After 20 days of exposure: MCs and total chlorophyll in *B. oleracea* & *S. tuberosum*
**Treatment 4 (T4)**: Combined exposure: Raw dam water containing MC-LR: 10.47 ± 3.879 μg L ^-1^; MC-RR: 6.158 ± 4.127 μg L ^-1^; MC-YR: 8.160 ± 2.544 μg L ^-1^ and LAS (3.4 mg L ^-1^)	After 5 days of exposure: MCs in *B. oleracea* After 20 days of exposure: MCs and total chlorophyll in *B. oleracea* & *S. tuberosum*

### Determination of total chlorophyll

In this study, total chlorophyll was monitored to assess the well-being of the plants upon exposure to the treatments. Oxidative stress in higher plants is known to inhibit synthesis and buildup of chlorophyll ( Agathokleous *et al.*, 2020), thus the estimation of total chlorophyll has been commonly used to indirectly monitor reactive oxygen species (ROS) in the plants ( Venkidasamy *et al.*, 2019).

Chlorophyll content was measured according to Baskar *et al*. (2015). 50 mg of the leaves were crushed using a mortar and pestle, and then soaked in 10 mL of 95 % (v/v) ethanol, and kept at room temperature in the dark for 72 hours. This was then followed by centrifuging for 30 minutes at 2264 × g, then collection of the supernatant and reading of the absorbance at 664.2 and 648.6 nm using a UV-vis spectrophotometer (SPECTROstar Nano, BMG LABTECH, Germany). Equations 1, 2 and 3 (below) were then used to calculate the chlorophyll- *a* and *b*, and the total chlorophyll content.

Chla=13.36A664.2−5.19A648.6 [1]

Chlb=27.43A648.6−8.12A664.2 [2]

Total chlorophyll=Chla+Chlb. [3]

Total chlorophyll content was expressed as milligram per gram per fresh matter (FM).

### Determination of LAS in water samples

LAS in the water samples was quantified using the methylene blue active substances test kits supplied by HANNA Instruments (South Africa). The test kits uses a colometric technique, based on methylene blue to detect and quantify anionic surfactants in 25 mL of the water sample. A HANNA HI96769 Anionic Surfactant Portable Photometer (HANNA Instruments, South Africa) was used to get readings of the levels of LAS in the samples. The assay was conducted according the manufacturer’s instructions which come with the kits.

### Quantification of cyanotoxins in plant material

Microcystins (MC-LR, MC-RR, and MC-YR) in plant tissues were determined using a triple quadrupole LC–MS/MS system (model 8045, Shimadzu Corporation, Japan). Toxin extraction was conducted using a modification of the method used by Manubolu *et al*. (2018). Accurately weighed, 100 g of plant material (leaves for *B. oleracea* and tubers for *S. tuberosum*) were freeze-dried for 48 hours at −48 ^o^C under a constant vacuum of 44 μmHg (Telstar Lyoquest Freeze Dryer, Terrassa, Spain). The freeze-dried material was then ground to powder using a mortar and pestle.

10 mL of 50% methanol solution was added to 1 g of each freeze-dried sample and sonicated for 5 minutes in a water bath (SCIENTEC Ultrasonic Cleaner, Model 705, South Africa) for 5 minutes. Upon sonication, the plant extracts were then centrifuged for 30 minutes at 2264 × *g.* The whole process of sonication, centrifugation, and collecting the supernatant was repeated thrice and the supernatant was pooled to give approximately a 30 mL extract of each sample. The plant extract (30 mL) and water samples (100 mL) were cleaned up using solid phase extraction (SPE) with HLB (3 cc, 60 mg, Waters Oasis).

For SPE with HLB, the cartridges were first conditioned with methanol (6 mL), followed by ultrapure water (6 mL). The samples (± 30 mL for extracts and 100 mL water) were slowly loaded onto the cartridges, followed by rinsing with 20% methanol. The cartridges were then eluted with 10 mL of 80% methanol. Lastly, the eluent was then dried at 50 ^o^C under a stream of nitrogen (N _2_) gas. The dried samples were reconstituted in 1 mL of 80% methanol prior to LC-MS/MS analysis.

*Chromatographic conditions*

Levels of microcystins (-LR, -RR and -YR) in the water samples and plant extracts were determined on a triple quadrupole LC–MS/MS system (model LCMS-8045, Shimadzu Corporation, Japan) with a Shim-pack Velox SP-C18, 2.7 μm, with dimensions 2.1 × 100 mm (Shimadzu, Japan). The injection volume was set at 10 μL and the mobile phases used were 0.1% formic acid (FA) in water (A) and 0.1% FA in acetonitrile (B). A flow rate of 0.4 mL min ^-1^ and a 5-minute binary gradient was used with an elution profile of: 2% B (0.4 min), linear gradient to 70% B (3.1 min), 100% B (0.5 min), and, finally, 2% B (1 min).

The LC-MS/MS interface conditions were: 300 ^o^C interface temperature, 3 L min ^-1^ for the nebulizing gas flow, 235 ^o^C DL temperature, 10 L min ^-1^ for both drying gas and heating gas flow and interface voltage of 3.0 kV for electrospray in the positive (ES+) mode.

**Figure 1.**
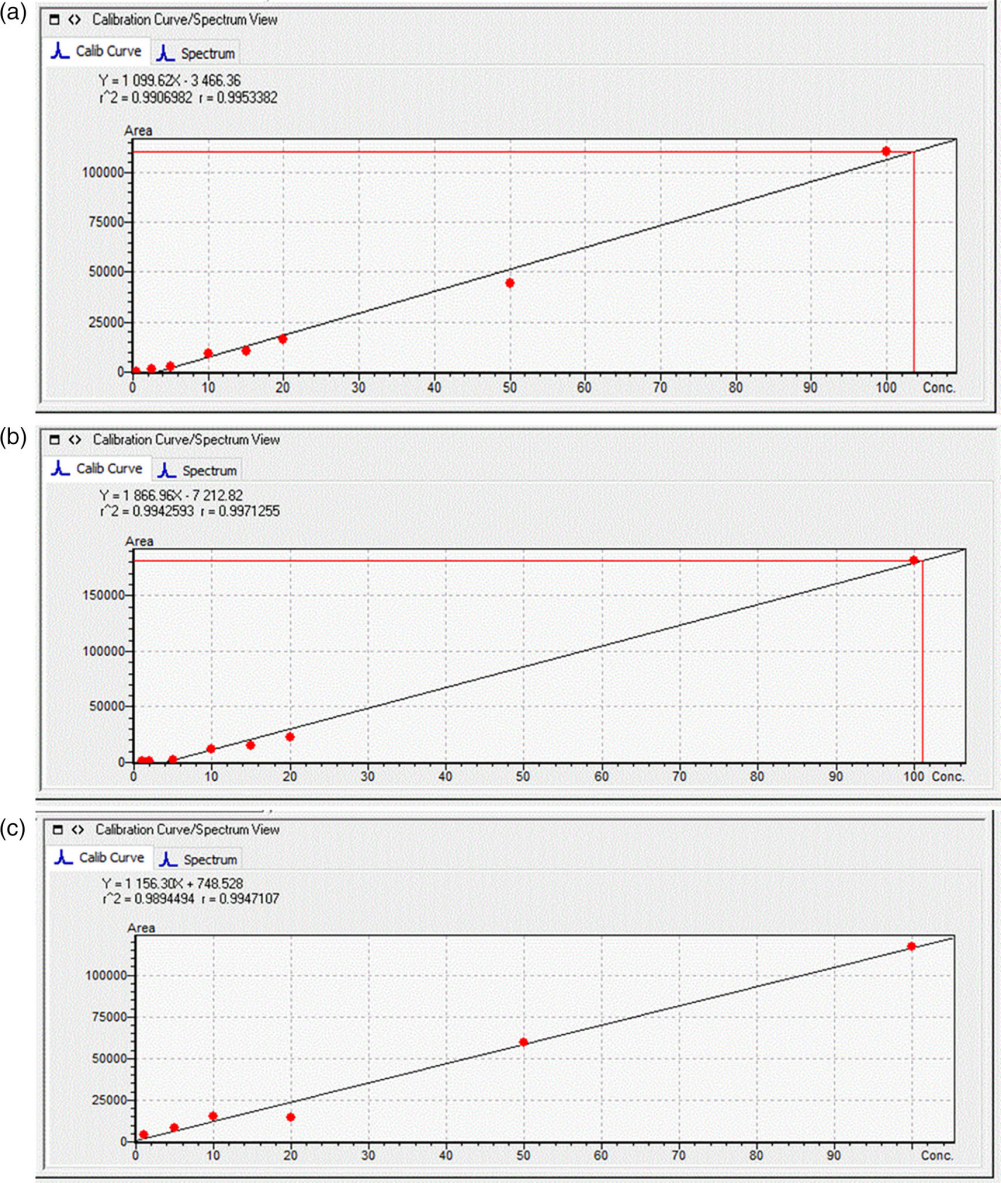
Calibration curves obtained for (a) Microcystin-RR (MC-RR); (b) MC-YR; and (c) MC-LR; in *B. oleracea* leaf extract.

**Figure 2.**
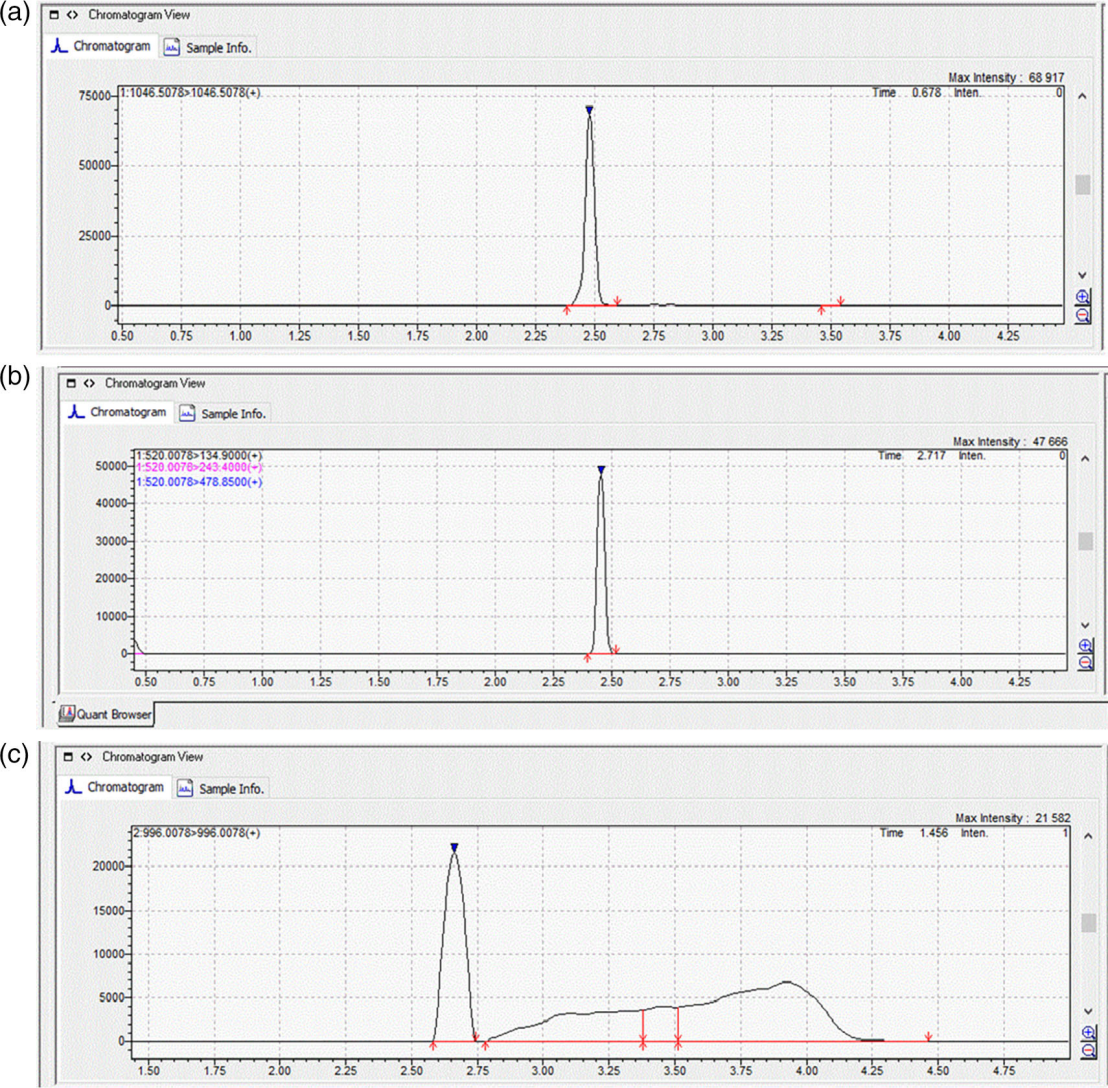
MRM chromatograms of quantification ions for the three MCs, (a) MC-RR; (b) MC-YR and (c) MC-LR at a concentration of 100 μg L ^-1^.

To quantify the individual congeners of MCs, an external standard quantitation technique was used. Standard solutions at seven different concentrations (1, 2, 5, 10, 20, 50, and 100 μg L ^-1^) were prepared using cabbage leaves extracts and potato tuber extracts and these were used to quantify the toxins in the plant samples ( Figure 1). The MRM chromatograms of the quantification ions for the three MCs at a concentration of 100 μg L ^-1^ are shown in Figure 2.

The final concentration of toxins in each sample was determined using equation 4:

$$\text{Conc in sample}\;\left(\mathrm{\mu g}\;L^{-1}\right)=\left(\frac{C_{o}\times \text{Vol of extract used}\;\left(L\right)}{\text{Volume of sample used}\;\left(L\right)}\right)$$ [4]

Where $C_{o}$ = the concentration of the sample determined from the calibration curve (μg L ^-1^).

### Estimated daily intake (EDI)

Equation 5 was utilized to estimate the daily intake of cyanotoxins for an average size human ( Bartos, 2020):

$$\text{EDI}=\frac{T\times C}{M}$$ [5]

Where:

*T* = the concentration of toxins in the edible fractions of the cabbage ( *T*, μg kg ^-1^ fresh mass).

*C* = the daily consumption amounts of cabbage ( *C*, kilograms per day).

*M* = mass of an average-sized human ( *W*, 60 kg adult).

N.B: It was assumed that the consumption of cabbage is similar to that of lettuce and used 85 g dry mass (DM) of cabbage and 148 g dry mass (DM) of potatoes based on the U.S. FDA (2017) suggested serving size ( Bartos, 2020). An EDI of >0.04 exceeds the total daily intake limit set by the WHO.

### Data analysis

To compare the levels of accumulated MCs and the total chlorophyll of the various plant treatments, analysis of variance (ANOVA) and/or the Kruskal-Wallis tests were used at 95% confidence intervals (CI) using GraphPad InStat 3 (GraphPad Software, California, United States; RRID:SCR_000306). Kolmogorov–Smirnov and Bartlett tests were used to test for normality and variance homogeneity at 95% CI. Data which passed this test was compared using ANOVA while data which did not pass the test was compared using Kruskal–Wallis at 95% CI. The Tukey-Kramer multiple comparisons test and Dunn’s multiple comparisons test were used as *post-hoc* assays for data which passed the normality tests and data which did not pass the normality test, respectively. Levels of MCs are presented by their means ± the standard deviation (SD).

## Results & discussion

### Physicochemical parameters of the dam water

The dam water used to water the plants was slightly alkaline, with a mean pH of 9.02 ± 0.29, had high EC and TDS levels (380 ± 16.52 μS cm ^-1^ and 228 ± 7.51 mg L ^-1^, respectively). The water also had a high algal biomass (Chlorophyll- *a* 440.24 ± 328.147 μg L ^-1^). The raw dam water had the following mean concentration ± standard deviation of MC-LR: 10.47 ± 3.879 μg L ^-1^; 6.158 ± 4.127 μg L ^-1^ for MC-RR, and 8.160 ± 2.544 μg L ^-1^ for MC-YR. The pH of the water was above the 6.5–8.4 threshold for water intended for irrigation in S. A ( DWAF, 1996). Even though the EC of the dam water was quite high, it was within the S. A ( DWAF, 1996) guideline and the FAO (1985) ( Ayers and Westcot, 1985) limits for irrigation water, which are set at ≤400 μS cm ^-1^ and 700 μS cm ^-1^, respectively. The levels of anionic surfactants in the water ranged from 0.13 to 3.4 mg L ^-1^.

### Bioaccumulation of cyanotoxins in *S. tuberosum* and *B. oleracea*

To identify and quantify MCs, two multiple reaction monitoring (MRM) transitions for MC-LR and MC-YR were selected and optimized, with the most abundant ionic product utilized for quantitation and the other for confirmation, whereas for MC-RR only one transition was used. For MC-LR and MC-YR, the single protonated molecular ions [M + H] ^+^ were formed, as a result of the presence of one arginine moiety, which is the most preferred protonation site for these compounds ( Zervou *et al*., 2017). The MRM transitions employed for MC-LR were 996.0078/996.00 and 498.5078/162.90; for MC-RR: 520.0078/134.90; for MC-YR: 1046.5078/1046 and 523.7578/127.00, with the first one being used for quantification and the second one for confirmation (for MC-LR and MC-YR).

With respect to all the three MCs monitored, the mass-to-charge ratio (m/z) signal 135 was the main ionic product. Like other polypeptides, MCs form sodium replacement ions which results in ion envelopes at each charge state apparent in mass spectra ( Draper *et al*., 2013). For MC-RR the transition with a m/z of 520.0078 corresponds to the double charged protonated molecular ion [M + 2H] ^2+^ precursor ions, as they contain two arginine residues in their molecular structure ( Draper *et al*., 2013; Zervou *et al*., 2017).

### Bioaccumulation of cyanotoxins in *S. tuberosum*

When exposed to the four different treatments for a period of 20 days, Table 2 shows the mean ± SE concentrations of MCs accumulated in the tubers of *S. tuberosum.* The accumulation patterns resembled the levels of MCs in the raw water samples, with plants exposed to raw dam water ( *T2* and *T4*) showing higher levels of MC-LR, followed by MC-RR then MC-YR. Statistically significant differences among the mean levels of accumulated toxins were reported for MC-LR and MC-YR, whereas MC-RR did show any statistically significant differences (ANOVA/Kruskal-Wallis Test, at 95% CI) among the four treatments. Higher levels of the toxins accumulated in plants exposed to raw dam water ( *T2*) compared to the other three treatments. The presence of LAS in the raw dam water in *T4* did not result in higher uptake and accumulation as anticipated.

**Table 2.** Mean (±SE) MCs accumulated in *S. tuberosum* tubers upon 20-day exposure to the four treatments.

	T1: Control (Milli-Q water)	T2: Roodeplaat Dam water	T3: Milli-Q water with LAS	T4: Roodeplaat Dam water with LAS	*P*-value	EDI (mg kg ^−1^ of body mass day ^−1^)
**MCRR** (ng g ^−1^ DW)	0.253 (±0.173) ^a^	4.851 (±1.194) ^a^	0.535 (±0.272) ^a^	3.820 (±1.481) ^a^	0.079 (n.s)	0.007
**MCYR** (ng g ^−1^ DW)	0.554 (±0.178) ^a^	6.404 (±2.618) ^a,b^	1.167 (±0.843) ^b^	5.309 (±1.812) ^a,c^	0.001 ***	0.009
**MCLR** (ng g ^−1^ DW)	16.041 (±11.900) ^a^	200.387 (±66.200) ^b^	22.709 (±11.930) ^a,c^	150.868 (±60.600) ^a,b,d^	0.001 **	0.284 ^†^

Data labelled with different superscripts (a–d) differed significantly in each row.

* *p* < 0.05.

** *p* < 0.01.

*** *p* < 0.001, n.s = not significant.

^†^ denotes data which exceeded the EDI.

Except for MC-LR, the levels of MCs accumulated by the tubers did not exceed the TDI of 0.04 mg kg ^-1^ of body mass recommended by the WHO. Since MC-LR is normally the dominant congener in many waters dominated by the *Microcystis* and *Aeruginosa* genera, the raw dam water used was dominated by MC-LR, hence it was the only congener which exceeded the recommended TDI.

### Bioaccumulation of cyanotoxins in *B. oleracea*

Regarding the accumulation of the toxins in *B. oleracea* leaves, Table 3 and Table 4 show the mean levels of MCs accumulated in the leaves of the plants after 5 days and 20 days, respectively. Based on the findings, a clear increase in the accumulation of the three MCs in the plants from the 5 ^th^ day to the 20 ^th^ day is observed. Statistically significant differences in treatments were observed for MC-YR and MC-LR after 5 days of exposure, with significantly higher accumulations observed in *T2* followed by *T4,* compared to the other treatments (ANOVA/Kruskal-Wallis Test at p = 0.05).

**Table 3.** Mean (±SE) MCs accumulated in *B. oleracea* leaves upon 5-day exposure to the four treatments.

	T1: Control (Milli-Q water)	T2: Roodeplaat Dam water	T3: Milli-Q water with LAS	T4: Roodeplaat Dam water with LAS	*P*-value	EDI (mg kg ^−1^ of body mass day ^−1^)
**MCRR** (ng g ^−1^ DW)	0.583 (±0.233) ^a^	11.646 (±8.315) ^a^	0.884 (±0.433) ^a^	12.962 (±4.636) ^a^	0.543 (n.s)	0.018
**MCYR** (ng g ^−1^ DW)	0.590 (±0.116) ^a^	5.786 (±0.712) ^b^	0.603 (±0.082) ^a^	5.473 (±0.698) ^b^	< 0.000 ***	0.008
**MCLR** (ng g ^−1^ DW)	0.011 (±0.015) ^a^	3.271 (±1.531) ^b^	0.027 (±0.016) ^a^	1.076 (±1.064) ^a^	< 0.000 ***	0.005

Data labelled with different superscripts (a–d) differed significantly in each row.

* *p* < 0.05.

** *p* < 0.01.

*** *p* < 0.001, n.s = not significant.

**Table 4.** Mean (±SD) MCs accumulated in *B. oleracea* leaves upon 20-day exposure to the four treatments.

	T1: Control (Milli-Q water)	T2: Roodeplaat Dam water	T3: Milli-Q water with LAS	T4: Roodeplaat Dam water with LAS	*P*-value	EDI (mg kg ^−1^ of body mass day ^−1^)
**MCRR** (ng g ^−1^ DW)	0.155 (±0.069) ^a^	83.923 (±29.750) ^b^	0.683 (±0.362) ^a^	34.973 (±21.450) ^a,b^	0.014 *	**0.119** ^†^
**MCYR** (ng g ^−1^ DW)	0.630 (±0.116) ^a^	6.938 (±0.547) ^b^	0.521 (±0.040) ^a^	5.134 (±0.842) ^c^	< 0.000 ***	0.010
**MCLR** (ng g ^−1^ DW)	0.022 (±0.017) ^a^	5.741 (±1.303) ^b^	0.014 (±0.016) ^a^	2.531 (±1.939) ^c^	< 0.000 ***	0.008

Data labelled with different superscripts (a–d) differed significantly in each row.

* *p* < 0.05.

** *p* < 0.01.

*** *p* < 0.001, n.s = not significant.

^†^ denotes data which exceeded the EDI.

Findings in Table 4 show that statistically significant differences were found among the treatments for all of the three congeners of MCs (ANOVA/Kruskal-Wallis Test at *p* = 0.05) in *B. oleracea* leaves upon 20 days of exposure to the four treatments. Similar to the patterns observed for *S. tuberosum* tubers and for *B. oleracea* leaves after 5-day exposures, higher levels of MCs accumulated in *T2*, followed by *T4,* compared to the other treatments. The findings imply that the presence of LAS in *T3* did not have any impact on the uptake of MCs from the soil (without exposure to MCs in irrigation water) and that the presence of LAS in raw dam water in *T4* did not enhance the uptake and accumulation of MCs by the plants.

Much of the work on the combined ecotoxicological risks of LAS and MCs has been done by Wang *et al*. (2011, 2012). According to Wang *et al*. (2011), LAS affects organisms by altering their membrane permeability, the activity of enzymes, and the structure of tissues in organisms ( Wang *et al*., 2011). Contrary to our findings, where the presence of LAS did not impact the accumulation of MC-LR in plants, Wang *et al*. (2011) reported higher accumulation rates when lettuce seedling were exposed to a combination of MC-LR and LAS compared to MC-LR alone.

Similar to our findings, where we found higher levels of MCs in potato tubers compared to cabbage leaves, Wang *et al*. (2011) reported higher levels in roots compared to other parts of the plants. In contrast to our findings, Wang *et al*. (2012) found enhanced uptake of MC-LR in duckweed even at the lowest concentration of 3 μg mL ^-1^, which was comparable to the 3.4 μg mL ^-1^ used in this study. The major difference between these experiments was the media in which the experiments were conducted, with Wang *et al*. (2012) having used aquatic plants compared to the terrestrial plants tested in this study.

In the current study, the presence of microbes with the potential to degrade LAS in the soil could have been a major factor. According to Mao *et al*. (2015) at low concentrations, surfactants build up at the liquid to liquid or at the solid to liquid interface as monomers. Increasing their concentrations, eventually replaces the interfacial solvent, such as water, leading to decreased polarity of the aqueous-phase and a surface tension reduction. In high concentrations of surfactants, dissolved pollutants in the aqueous phase gain more mobility which is conducive for removal and uptake by plants and even degradation by microbes. In addition, the properties of the soil and the surfactant itself may influence the adsorption of a surfactant ( Mao *et al*., 2015). Previous studies have also demonstrated that soils have the potential to temporarily make cyanotoxins unavailable for uptake by plants through chemical and physical modification, though this is dependent on the type of soil ( Bartos, 2020).

The interaction and combination of LAS and other contaminants including microcystins and metal ions has been found to be both synergistic and, in some cases, antagonistic ( Chai *et al*., 2020). Our findings did not suggest any synergistic nor antagonistic effects of LAS in combination with MCs in the water used. Consistent to our findings, Zhang *et al*. (2008) did not find increased uptake of Cadmium (Cd) by soybeans in the presence of LAS. Jensen and Sverdrup (2002) also did not find any combined effect of LAS and pyrene on the *Folsomia fimetaria.* According to Chai *et al*. (2020) synergistic or combined effects are influenced by a number of factors including the types of contaminants tested, the plant species, the concentrations tested, and the duration of exposure. In this study factors such as faster biodegradation of LAS by microbes and the low concentrations of LAS tested could all have affected the activity and toxicity to the plants of LAS, MCs, and other contaminants.

Even though MC-RR was of a lower concentration in the raw dam water compared to MC-LR, the findings of the current study have shown that it can accumulate in cabbage leaves to levels which can exceed the 0.04 mg day ^-1^ kg ^-1^ of body mass when plants are watered with contaminated dam water. This is of concern since this limit was reached after only 5 days of exposure to the dam water. However the fact that the EDI was not exceeded in the cabbage leaves after 20 days of exposure to the same dam water could be a reflection that the plants were finding ways of coping and bio-transforming the toxins as the exposure was prolonged. Xiang *et al.* (2020) established that the biotransformation and depuration rates of MC-LR in plants can sometimes exceed its uptake and there can also be higher degradation in soils, thus lower bioaccumulation from soil.

It is also important to mention that the WHO has provisional TDI values for MC-LR only and not for other MC congeners and here we calculated EDIs for each of the three MC congeners monitored. Based on that, assuming that all the MCs have similar impacts on human beings, the combined EDIs for the MCs monitored here and those not monitored in this study can easily exceed the set TDI values.

### Effects of LAS and MCs on *B. Oleracea* and *S. tuberosum*

Since exposure to increased levels of MCs in plants is known to induce oxidative stress, hinder the plants’ ability to produce chlorophyll, and inhibit photosynthesis ( Campos *et al*., 2021), total chlorophyll levels in the two plant species were monitored to assess the potential effects of LAS and MCs on the plants. Monitoring of plant chlorophyll is a well renowned method for monitoring the well-being of plants since it correlates well with other parameters such as plant nitrogen, carotenoids and green fluorescence ( Cortazar *et al.*, 2015).

In addition to the two stressors (MCs and LAS), the dam water used in treatments *2* and *4* also had a high EC (380 ±16.52 μs cm ^-1^) and TDS levels (228 ±7.51 mg L ^-1^), indicating contamination with other salts. All these contaminants, for example MCs ( Saqrane *et al*., 2008; Machado *et al*., 2017), high pH, high EC ( Huang *et al*., 2017), and anionic surfactants ( Pandey & Gopal, 2010; Wang *et al*., 2012) are also known to induce oxidative stress, reduce chlorophyll production, and affect plant growth.

**Figure 3.**
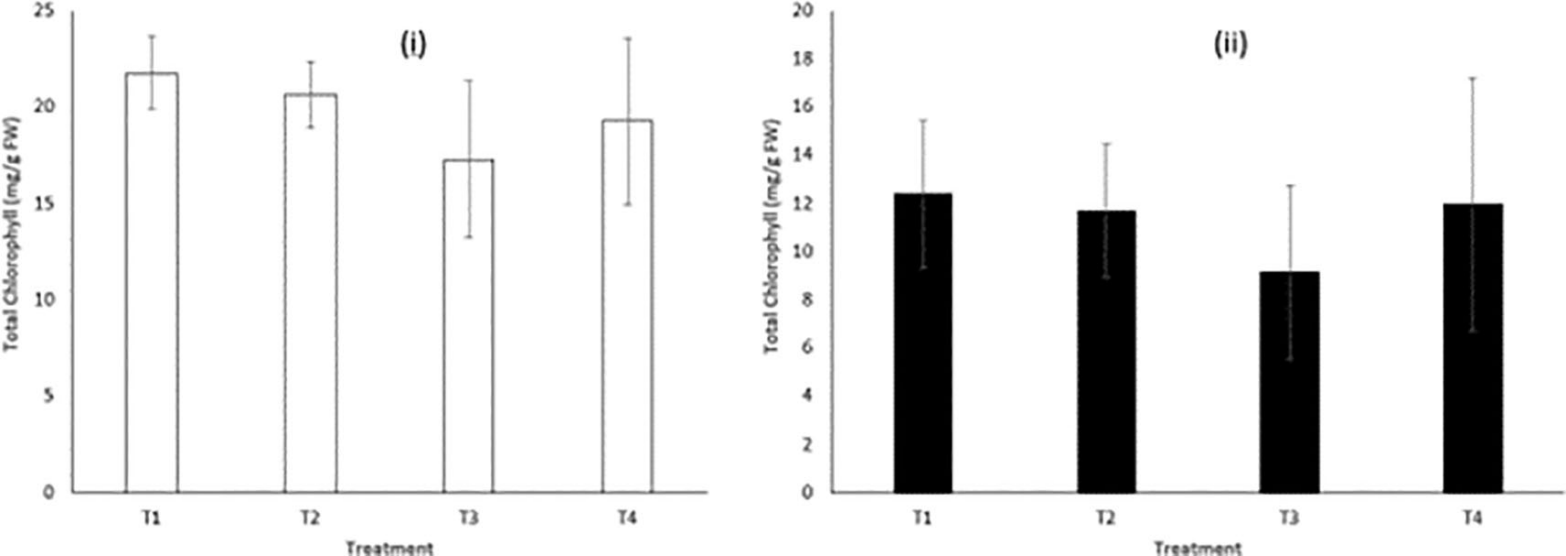
Total chlorophyll in (i) cabbage ( *Brassica oleracea*) leaves (ii) potato ( *Solanum tuberosum*) leaves, after 20 days of exposure to the four treatments.

Figure 3 (i) shows higher total chlorophyll content in the leaves of *B. oleracea* plants exposed to treatment *1* compared to other treatments. There were no statistically significant differences in the mean total chlorophyll content among the plants exposed to the four treatments after 20 days of exposure ( *p* > 0.05). This implied that the levels of MCs, LAS, and other pollutants in the raw dam water used were not high enough to impact the synthesis of chlorophyll and other photosynthetic processes in the plants.

Concerning the total chlorophyll content of the *S. tuberosum* leaves, higher total chlorophyll levels were observed in plants exposed to treatments *1* and *4*, but there were no statistically significant differences in the mean total chlorophyll levels among the four treatments ( *p* > 0.05). The findings imply that exposure to environmentally relevant levels of MCs and LAS, as applied in this study, and the high EC in the raw dam water did not induce oxidative stress nor inhibit chlorophyll production in the plants. In addition, no significant visual impacts were observed on the plants exposed to the four treatments.

Even though no symptoms of toxicity were reported in this study, MCs are known induce various subcellular effects which in turn lead to cell death in plants ( Máthé *et al.*, 2021). Cell deaths and other non-specific effects have been reported when plants are exposed to high concentrations of MCs which in most cases are not environmentally-relevant, whereas low level exposure to the toxins can cause a range of stimulatory consequences such as mitosis ( Máthé *et al.*, 2021) and inhibition of the protein serine/threonine phosphatases (PP2A/PP1) in cells ( Máthé *et al.*, 2021; Roué *et al.*, 2018).

Pappas *et al.* (2022) reported disruption of the main glucan, protein and pectic components of plant cell walls upon exposure to crude extracts from cyanobacteria. Lignification of root tissue in higher plants upon exposure to various levels of MC-LR have has been reported in various plant species like the white mustard ( *Sinapis alba*) and the common reed ( *Phragmites australis*). Lignification upon exposure to high temperatures, heavy metals, salinity and lack of water is known to be a common mechanism for coping with stress in higher plants ( Pappas *et al.*, 2022).

## Conclusions

Based on the findings, the presence of the anionic surfactant (LAS) did not induce or promote the uptake of MCs by the two plant species. In addition, the presence of LAS and MCs in the irrigation water did not affect the total chlorophyll content and well-being of the tested plants. The study demonstrated that irrigation of terrestrial food plants with cyanobacteria-infested water from dams, such as Roodeplaat, can lead to MCs accumulating in the edible parts of the plants to levels that can exceed the set TDI of 0.04 mg kg ^-1^ of body mass, as recommended by WHO. Long-term studies investigating the levels of cyanotoxins in irrigation water in areas such as the Crocodile (West) Marico WMA and their potential impacts on crop productivity and the dietetic acceptability of such plants for human consumption are recommended. Such studies will need to factor in the local climate, soil types, degradation rates of cyanotoxins in the soil, and also consider a variety of cyanotoxin classes for a proper risk assessment.

## Author contributions

**Pindihama G.K.**: Conceptualization, Methodology, Data curation Roles/Writing - original draft; Writing - review & editing, Investigation; Methodology.

**Gitari W.M.**: Conceptualization, Methodology, Data curation Roles, Supervision; Funding acquisition; Investigation; Resources; Roles/Writing - original draft; Writing - review & editing.

**Madala N.E.**: Data acquisition/sample analysis, Writing - review & editing.

## Data availability

### Underlying data

The knowledge network for biocomplexity: Effect of linear alkylbenzene sulfonate on the uptake of microcystins by *Brassica Oleracea* and *Solanum Tuberosum*, DOI: 10.5063/F16M3589 ( Pindihama, 2022).

This project contains the following underlying data:

- Data file 1: comparison of MC levels in the two plants
- Data file 2: total chlorophyll data for *Brassica oleracea*
- Data file 3: total chlorophyll for *Solanum tuberosum*

Data are available under the terms of the Creative Commons Zero “No rights reserved” data waiver (CC0 1.0 Public domain dedication).
