# Supplementary material for: Effect of linear alkylbenzene sulfonate on the uptake of microcystins by Brassica oleracea and Solanum tuberosum
Source: F1000Res. 2024 Jan 26;11:1166. Originally published 2022 Oct 12. [Version 2] doi: 10.12688/f1000research.125540.2 (PMC10951562; doi:10.12688/f1000research.125540.2)
Supplement: Peer review history

# Reviewer report: Reviewer response for version 2

**DOI:** 10.5256/f1000research.161360.r241026

**Published:** 2024-03-19

**Authors:** Rialet Pieters

**Affiliations:** 1 Unit for Environmental Sciences and Management, North-West University, Potchefstroom, South Africa

I am satisfied with the current version of the publication.

Is the work clearly and accurately presented and does it cite the current literature?

Partly

If applicable, is the statistical analysis and its interpretation appropriate?

Yes

Are all the source data underlying the results available to ensure full reproducibility?

No

Is the study design appropriate and is the work technically sound?

No

Are the conclusions drawn adequately supported by the results?

Partly

Are sufficient details of methods and analysis provided to allow replication by others?

No

Reviewer Expertise:

Ecotoxicologist with a good understanding of what is required in a scientific paper containing analytical chemistry data.

I confirm that I have read this submission and believe that I have an appropriate level of expertise to confirm that it is of an acceptable scientific standard.

# Reviewer report: Reviewer response for version 2

**DOI:** 10.5256/f1000research.161360.r241027

**Published:** 2024-02-21

**Authors:** Ioannis-Dimosthenis S Adamakis

**Affiliations:** 1 Section of Botany, Department of Biology, National and Kapodistrian University of Athens, Athens, Greece

I believe that the authors have adressed previous concerns

Is the work clearly and accurately presented and does it cite the current literature?

Partly

If applicable, is the statistical analysis and its interpretation appropriate?

I cannot comment. A qualified statistician is required.

Are all the source data underlying the results available to ensure full reproducibility?

Yes

Is the study design appropriate and is the work technically sound?

No

Are the conclusions drawn adequately supported by the results?

No

Are sufficient details of methods and analysis provided to allow replication by others?

No

Reviewer Expertise:

Plant stress biology

I confirm that I have read this submission and believe that I have an appropriate level of expertise to confirm that it is of an acceptable scientific standard.

# Reviewer report: Reviewer response for version 1

**DOI:** 10.5256/f1000research.137858.r182592

**Published:** 2023-07-12

**Authors:** Ioannis-Dimosthenis S Adamakis

**Affiliations:** 1 Section of Botany, Department of Biology, National and Kapodistrian University of Athens, Athens, Greece

The subject that is being addressed is very interesting however there are some concerns which need to be addressed prior acceptance.

1. Hypereutrophic conditions leading to increased presence of microcystin producing bacteria is a problem not only in South Africa. Some statements in the manuscript makes it of topical interest and should be avoided.
2. Microcystins are toxic to plants. Even if you did not find any symptoms of toxicity you should discuss this. Below I have added some references.
3. The material and methods section is not adequate. A more detailed description of the methodology is needed. For instance, the authors state that the plants were watered with water for the dam where microcystins were found. How were those concentration measured? From which species of cyanobacteria?
4. The well being of plants are not only determined be the chlorophyll content. The determination of more parameters are needed to make such a statement.
5. The English needs polishing.

Is the work clearly and accurately presented and does it cite the current literature?

Partly

If applicable, is the statistical analysis and its interpretation appropriate?

I cannot comment. A qualified statistician is required.

Are all the source data underlying the results available to ensure full reproducibility?

Yes

Is the study design appropriate and is the work technically sound?

No

Are the conclusions drawn adequately supported by the results?

No

Are sufficient details of methods and analysis provided to allow replication by others?

No

Reviewer Expertise:

Plant stress biology

I confirm that I have read this submission and believe that I have an appropriate level of expertise to confirm that it is of an acceptable scientific standard, however I have significant reservations, as outlined above.

## References

[1] : Subcellular Alterations Induced by Cyanotoxins in Vascular Plants-A Review. *Plants (Basel)* .2021;10(5) : 10.3390/plants10050984 10.3390/plants10050984 34069255 PMC8157112

[2] : Microcystin-LR and cyanobacterial extracts alter the distribution of cell wall matrix components in rice root cells. *Plant Physiol Biochem* .2022;191: 10.1016/j.plaphy.2022.09.020 78-88 10.1016/j.plaphy.2022.09.020 36195035

# Reviewer report: Reviewer response for version 1

**DOI:** 10.5256/f1000research.137858.r153265

**Published:** 2022-11-07

**Authors:** Rialet Pieters

**Affiliations:** 1 Unit for Environmental Sciences and Management, North-West University, Potchefstroom, South Africa

Summary: The possibility of LAS enhancing uptake of MC from water and soil into 2 vegetables was investigated. Seedlings were treated with uncontaminated water, water with LAS, water with MCs, and water with both MCs and LAS. 

 Issues to be addressed:

- Use 'mass' in all instances where the unit is ug or g or kg and not 'weight'. The SI unit for weight is Newton. E.g., body mass, dry mass and molecular mass.
- Elimination of LAS in Intro "occurs via adsorption and biodegradation". Elaborate onto what LAS may adsorb with references.
- "This makes hypereutrophic lakes and reservoirs ideal environments for the co-existence of toxic cyanobacteria since" insert 'and LAS' between 'cyanobacteria' and 'since'.
- "Previous studies have reported enhanced MCs" Remove 'have'
- "combination with other stressors including LAS, on terrestrial food plants is of importance." Insert 'to the humans consuming these plants' after 'importance'.

Issues with methods:

- Analysis of LAS never described.
- Only nominal concentrations of MCs in treatments, and not measured concentrations which are crucial.
- No internal standards were used in the analysis of plants for MCs. Therefore, no determination of recoveries.
- "The farm lies in the low veld climate..." Lowveld
- "...respectively. All of which indicated..." , all
- "...total daily intake limit set by WHO" Insert 'the' after 'by'.
- "...15 kg of the soil was placed into 350mm plastic pots for..." This, 350 mm, is only one length. The pots are likely to have volume and this should be reported.
- "...the media were tested daily and refreshed accordingly". This description is too vague. At which depths were the soil media sampled? And how was it 'refreshed'? By removing the plant from the soil and replacing with newly prepared media containing concentrations of which? Or is the media referring to 'dam water'? If this is the case, what is meant by 'refreshing the media/water?
- "Chlorophyll content was measured..." What is this a measure of? In other words, why would you want to determine this?
- "...followed by incubation in the dark for 72 hours."  'incubation' usually associated with temperature?
- "The water also had a high cyanobacterial biomass (Chlorophyll-a 440.24 +- 328.147 μg L-1)." Chlorophyll a is a measure of ALL algal mass, not only cyanobacterial (Biogeosciences Discuss., 4, 707–745, 2007).
- "Standard solutions at seven different concentrations (1, 2, 5, 10, 20, 50, and 100 μg L-1) were prepared using cabbage leaves extracts and potato tuber extracts and these were used to quantify the toxins in the plant samples (Figure 1). The MRM chromatograms of the quantification ions for the three MCs at a concentration of 100 μg L-1 are shown in Figure 2." Should move to M&M section.
- "...water) and that the presence of LAS in raw dam water in T4 did not enhance..." The quantification of this was not demonstrated in the preceding sections of the paper
- Suggestion: Report data in tables 2-4 in bar graphs. Will make for an easier comparison of results. Explain treatments again, or name them more descriptively.
- Report SD and not SE in tables 2-4. 
- Express concentration of Table 2-4 in ng/g to reduce zeroes after decimal point.
- "...used. Consistent to our findings, Zhang et al. (2008) did not find increased uptake of Cadmium..." No metals are reported in Results? And no analysis thereof is described in M&M. Rephrase this so that it becomes clear that you use the Cd as an example of a toxicant that similarly to MC is not taken up more by plants when in the presence of LAS
- "...plants were finding ways of copying and bio-transforming..." coping rather than copying.
- 'It is also important to mention that WHO has...' the WHO.
- Fig 3 legend:  "a indicates no significant difference among treatments (p > 0.05)."  Remove this sentence and the a's from the figure. You should really only indicate when there was stat. sig. difference like in tables 2-4 and not when there NOT stat. sig.
- "...the high EC in the raw dam water did not induce oxidative stress nor inhibit..." The preceding text in the manuscript does not show any evidence of the quantification of oxidative stress. This claim is therefore not supported by the data in the manuscript and should be removed.

Is the work clearly and accurately presented and does it cite the current literature?

Partly

If applicable, is the statistical analysis and its interpretation appropriate?

Yes

Are all the source data underlying the results available to ensure full reproducibility?

No

Is the study design appropriate and is the work technically sound?

No

Are the conclusions drawn adequately supported by the results?

Partly

Are sufficient details of methods and analysis provided to allow replication by others?

No

Reviewer Expertise:

Ecotoxicologist with a good understanding of what is required in a scientific paper containing analytical chemistry data.

I confirm that I have read this submission and believe that I have an appropriate level of expertise to state that I do not consider it to be of an acceptable scientific standard, for reasons outlined above.
